# High Prevalence of Food Insecurity and Associated Risk Factors in Chilean and Immigrant Women from South-Central Chile

**DOI:** 10.3390/foods14223973

**Published:** 2025-11-20

**Authors:** Alejandra Rodríguez-Fernández, Juana María Delgado-Saborit, Paula Carrasco, Gabriela Cormick, Marcela Ruiz-de la Fuente, Eduard Maury-Sintjago

**Affiliations:** 1 Departamento de Nutrición y Salud Pública, Universidad del Bío-Bío, Chillán 3780000, Chile; marcelaruiz@ubiobio.cl; 2 Grupo de Investigación en Auxología, Bioantropología y Ontogenia, Facultad de Ciencias de la Salud y de los Alimentos (FACSA), Universidad del Bío-Bío, Chillán 378000, Chile; 3 Departamento de Medicina, Universitat Jaume I, 12071 Castellón de la Plana, Spain; delgado@uji.es (J.M.D.-S.); pcarrasc@uji.es (P.C.); 4 Facultad de Ciencias de la Salud, Universidad Adventista de Chile, Chillán 378000, Chile; 5 Unidad Mixta de Investigación en Epidemiología y Salud Ambiental, FISABIO-Universitat Jaume I-Universitat de Valencia, 12071 Valencia, Spain; 6 Centro de Investigación Biomédica en Red en Epidemiología y Salud Pública (CIBERESP), Instituto de Salud Carlos III, 12071 Madrid, Spain; 7 Centro de Investigaciones en Epidemiología y Salud Pública (CIESP), Consejo Nacional de Investigaciones Científicas y Técnicas (CONICET), Buenos Aires 1414, Argentina; gabmick@yahoo.co.uk; 8 Instituto de Efectividad Clínica y Sanitaria (IECS), Consejo Nacional de Investigaciones Científicas y Técnicas (CONICET), Buenos Aires 1414, Argentina; 9 Departamento de Ciencias de la Salud, Universidad Nacional de la Matanza, San Justo 1754, Argentina

**Keywords:** food insecurity, women, immigrants, socioeconomic determinants

## Abstract

Food insecurity (FI) is a major public health problem that disproportionately affects women, especially if they are migrants. In Chile, there is limited data on how gender and migration status intersect to explain vulnerability to FI. A cross-sectional analytical study was conducted among 2124 women of childbearing age (1062 Chilean and 1062 immigrants) residing in south-central Chile. Biosociodemographic variables were collected through a structured questionnaire, and FI was assessed using the Household Food Insecurity Access Scale (HFIAS). Multivariate logistic regression models were applied to estimate risk factors using odds ratios (OR). Overall, 39.2% of women experienced some degree of FI, with prevalence significantly higher among immigrants (49%) compared to Chileans (29%). Severe FI was twice as frequent in immigrant women (18.1% vs. 9.2%). The risk factor of FI in the total sample included immigrant status (OR = 2.61; 95% CI: 2.15–3.17), low socioeconomic status (OR = 2.25; 1.77–2.87), having children (OR = 1.82; 1.49–2.23), being head of household (OR = 1.53; 1.25–1.87), not having a job (OR = 1.27; 1.02–1.58), and suffering from depression (OR = 2.11; 1.66–2.67). Subgroup analyses confirmed similar determinants in both groups, with not having a job being relevant mainly for immigrants and age acting as a protective factor among Chileans. FI is highly prevalent among women in south-central Chile, particularly among immigrants. Structural determinants such as socioeconomic status, having children, being the head of the household, and depression increase vulnerability. Policies must integrate gender and migration perspectives, promoting access to adequate food, employment, childcare, and mental health support.

## 1. Introduction

In the last decade, Chile has witnessed an unprecedented transformation in its demographic dynamics as a result of the rapid growth of migration, with a 47% increase in the foreign population being reported between 2018 and 2023. This phenomenon has made the country one of the main migratory destinations in South America, particularly receiving people from Venezuela (38%), Peru (14%), Colombia (11%), Haiti (9.8%), Bolivia (9.4%), and Argentina (4.3%) [1].

Migration is not a homogeneous experience, and gender roles play a crucial part in setting up migration paths and challenges. The so-called “feminization of migration” is not a neutral phenomenon: socially constructed gender norms often place migrant women in highly precarious occupations, such as domestic, care, and service work, with a dual role of productive and reproductive burden [2]. This overload increases women’s vulnerability to various forms of social exclusion, including food insecurity (FI), forcing them to prioritize the nutritional needs of the household over their own [3,4].

Food insecurity, defined by the FAO as the lack of secure access to sufficient, safe, and nutritious food for normal growth and development and for leading an active and healthy life, is currently one of the most pressing challenges in Latin America and the Caribbean, and it continues to affect different population groups unequally, especially at-risk groups such as women and migrants. The State of Food Security and Nutrition in the World report estimates that between 713 and 757 million people suffered from hunger in 2023, and that 28.9% of the world’s population experienced moderate or severe food insecurity. Latin America and the Caribbean approached this global average with 28.2%, above Asia (24.8%) and Oceania (26.8%) [4].

Several studies have documented that women often have the primary responsibility for their household’s diet, including food planning and purchasing, as well as daily preparation. This work, historically associated with the female role, positions women as primary agents in maintaining household food security [5,6]. However, this responsibility exposes them to greater nutritional risks, especially in contexts of poverty, exclusion, or migration [7]. Globally, the gender gap in FI deepened in 2022: 27.8% of women faced moderate or severe food insecurity, compared to 25.4% of men [4].

In Chile, despite the sustained increase in the migration phenomenon, public policies and health information systems have failed to fully incorporate variables such as nationality or country of origin. For instance, the National Health Survey (NHS) allows for the development of epidemiological profiles by sex, but not by migratory origin, which makes it difficult to specifically analyze living conditions and nutritional health of foreigners residing in the country [8].

Structural determinants faced by migrant women, such as informal employment, the high cost of living, inflation, social barriers, and the lack of access to support networks, directly affect their access to adequate food [9,10,11]. This situation is exacerbated when livelihood strategies, such as remittance transfers or relying on informal sector jobs, are not complemented by social protection policies that consider their gender and mobility trajectories. Added to this is the limited incorporation of gender-specific approaches into migration policies at global and local levels [12]. According to the IOM’s Migration Governance Indicators (MGIs), only 23% of countries worldwide integrate a gender perspective into their national migration strategies. This situation hinders equitable access to rights and opportunities, perpetuating forms of structural exclusion that particularly affect migrant women [13].

In this context, it is necessary to conduct research studies that incorporate a gender perspective into migration processes and food insecurity approaches, which have been poorly addressed, particularly in regions such as south-central Chile, where there is a convergence of factors such as social vulnerability, structural inequality, and cultural diversity. This study seeks to contribute to this effort, identifying risk factors associated with food insecurity in Chilean and immigrant women and providing evidence to guide public policies that are sensitive to gender and human mobility. Thus, the purpose of this research is to assess risk factors associated with food insecurity in immigrant and Chilean women in south-central Chile.

## 2. Materials and Methods

### 2.1. Design and Sample

This cross-sectional analytical study comprised 1062 immigrant women and an equal number of Chilean women of childbearing age. The immigrant group was mainly composed of Venezuelan women (49.6%), followed by participants from other countries (18.0%), such as Argentina (11.3%), Haiti (10.7%), Colombia (6.9%), and Peru (3.5%). Sample size calculation considered a simple random sampling stratified by the regions that constitute the south-central area of Chile (Ñuble, Bío-Bío, and Araucanía). Calculation considered an accuracy of 3% and a confidence level of 95%. Data collection took place between August 2022 and May 2023. Recruitment was conducted through a community-based field strategy using predefined geographic clusters within each region. The sampling frame consisted of municipal records and lists from local women’s organizations, which facilitated the identification of eligible women aged 18–49. Field teams invited participants through community health centers, municipal social programs, neighborhood associations, and community events. For comparative purposes, sample sizes were standardized across groups. The study included all migrants and Chileans who read and signed the informed consent form and voluntarily agreed to participate. Immigrant women were eligible only if they had been residing in Chile for up to five years at the time of data collection, ensuring that the sample represented recent migrants. Pregnant women and women on nutritional therapy were excluded.

The study was reviewed and approved by the Ethics and Biosafety Committee of the University of the Bío-Bío (Chile). All participants gave informed consent. The procedures were in accordance with the ethical standards of the Declaration of Helsinki and the Council of International Organizations of Medical Sciences [14].

### 2.2. Study Variables

Biosociodemographic characteristics were gathered through a questionnaire designed by the authors, where the following variables were recorded: age, group (Chilean/immigrant), having a partner (yes/no), having children (yes/no), being the head of household (yes/no), educational attainment (≥12 years/<12 years), and having a job (yes/no). Socioeconomic status was classified according to the ESOMAR socioeconomic system, which combines educational attainment and the occupational status of the main income earner [15]. For analytical purposes, categories A/B and C1 were grouped as high/medium-high SES, and categories C2, C3, and D/E were grouped as medium-low/low SES. Self-reported high blood pressure (HBP) (yes/no), type 2 diabetes mellitus (T2DM) (yes/no), high cholesterol (yes/no), depression (yes/no), and tobacco use (yes/no). All reported morbidities were assessed through a single self-reported item asking whether participants had ever been diagnosed with these conditions by a doctor or specialist.

Food insecurity: FI was assessed through the administration of the Household Food Insecurity Access Scale (HFIAS) for the Measurement of Food Access [16]. The main purpose of the HFIAS is to measure the prevalence of food insecurity. The HFIAS questionnaire consists of 9 questions, each of which is formulated in terms of remembering household access to food over the past 4 weeks. Finally, based on the respondent’s answers, the household can be classified as food secure or as having some level of food insecurity, which can be mild, moderate, or severe.

### 2.3. Statistical Analysis

The variables were described by absolute frequencies and percentages in the case of categorical variables, and by average and standard deviation in the case of numerical variables. Bivariate relationships were examined using the Chi-square statistical test. The modeling included multivariate logistic regression analysis by estimating crude and adjusted odds ratios (OR). A stepwise forward selection procedure was used in the multivariate logistic regression model, with variables entering at *p* < 0.05 and removed at *p* > 0.10. Model diagnostics included the evaluation of multicollinearity through variance inflation factors (VIF < 3.8), and goodness-of-fit was assessed using the Hosmer–Lemeshow test. Data processing was performed using the STATA v17 software (StataCorp LLC, College Station, TX, USA), considering a significance level of α < 0.05.

## 3. Results

The biosociodemographic description of the study participants is shown in Table 1. The average age of all the participating women was 32.5 ± 8.8 years. The distribution by age groups showed that most belonged to the 18–29 years group (39.9%), with this group also being more prevalent in Chileans (41.2%) compared to immigrants, where the age group 30–39 years was more prevalent, with 43.7% (*p* < 0.001). A total of 72.7% of women belonged to the lowest socioeconomic status categories, with immigrants accounting for a higher proportion with 75.7% compared to Chileans (69.7%) (*p* = 0.002). Regarding partner status, it was found that most women had a partner (65%), with Chileans showing a higher proportion (73.1%) than foreigners (56.9%) (*p* < 0.001). Having children was reported by 56.6%, with a similar proportion in both groups of women (*p* = 0.361). Regarding household headship, 49.1% reported being the head of the household, although a greater proportion was observed among foreign women (61%) as compared to Chilean women (40.8%) (*p* < 0.001). Educational attainment of ≥12 years was predominant (84.9%) and slightly higher in Chileans (86.8%) compared to foreigners (83.1%) (*p* = 0.015). Most women had a job (62.5%), although the percentage of employed immigrants was higher than their counterparts (68.9% and 56.1%, respectively) (*p* = <0.01). The prevalence of reported AHT was 22.4% of the total, being higher in immigrants than in Chileans (24.7% and 20.1%, respectively) (*p* = 0.013). A total of 19% of women had DMII, with similar proportions in both groups (*p* = 0.580), as well as the presence of high cholesterol, which reached a prevalence of 18.1% without differences by group (*p* = 0.693). A high prevalence (80%) of depression was found in all women, with this condition being higher in immigrants (84.8%) than in Chileans (74.2%) (*p* < 0.001). Tobacco use reached a prevalence of 22.9%, being higher in Chilean women (28%) compared to immigrant women (17.9%) (*p* < 0.001).

The prevalence of food insecurity reached 39.2% in all the women studied. Of this number, 14.7% were classified as mildly food insecure, 10.8% as moderately food insecure, and 13.7% as severely food insecure. When disaggregating FI by groups, it can be noticed that 49% of immigrant women experienced some degree of food insecurity compared to 29% of Chilean women. All FI levels were higher in immigrant women, with the category of severe food insecurity being twice as high as in Chilean women (18.1% and 9.2%) (Table 2).

To explain the variability of FI prevalence in all the women studied, with respect to the model, Table 3 shows an almost threefold increase in the risk of suffering FI in the case of immigrants as compared to Chilean women (OR = 2.61; 2.15–3.17). It can also be noticed that belonging to the lowest socioeconomic statuses increases to more than twice the risk of FI compared to the highest statuses (OR = 2.25; 1.77–2.87). Other factors that increase the risk of FI are having children (OR = 1.82; 1.49–2.23), being the head of household (OR = 1.53; 1.25–1.87), not having a job (OR = 1.27; 1.02–1.58), and having depression (OR = 2.11; 1.66–2.67) (Table 3).

When analyzing predictive factors for FI in each group of women, Table 4 shows that in immigrant women, the risk factors that predict food insecurity are associated with low socioeconomic status, which increases the risk of FI up to twice that observed in the highest statuses (OR = 1.95; 1.43–2.65). The same situation occurs if women have children (OR = 2.11; 1.62–2.75), are heads of household (OR = 1.39; 1.07–1.82), do not have a job (OR = 1.33; 1.01–1.83), and have depression (OR = 2.09; 1.44–3.05). On the other hand, risk factors that explain food insecurity in Chilean women are associated with increasing age, which is a protective factor with respect to younger women (OR = 0.97; 0.95–0.99). Risk factors were associated with low socioeconomic status, which increases the risk of FI by almost three times when compared to the highest status (OR = 2.94; 2.01–4.31). Similarly, women who have children are almost twice as at risk of experiencing FI (OR = 1.73; 1.18–2.54), as well as women who are heads of household (OR = 1.93; 1.42–2.79) and suffer from depression (OR = 1.83; 1.34–2.51) (Table 4).

## 4. Discussion

Sustainable Development Goal 2, Zero Hunger, aims to end all forms of hunger and malnutrition by 2030 and ensure access by all people to sufficient and nutritious food all year round [17]; therefore, estimating the prevalence of food insecurity in risk groups such as women and migrants is essential to make structural inequalities visible and provide evidence for the development of inclusive public policies in terms of public health.

Our results reveal that almost three out of ten Chilean participants suffer some degree of food insecurity, and the prevalence rises to a striking 49% when migratory trajectories are involved. These prevalences exceed the Latin American regional average (28.2%) and are twice the value of the most recent national prevalence based on the FIES (18.9%) [4,18]. The difference between food insecurity levels found in Chilean women and the national average can be explained by structural and territorial factors that differ between geographical areas. The Ñuble and Araucanía regions, both located in south-central Chile, have the highest incidence of income poverty in the country, with 12.1% and 11.6%, respectively, according to the 2022 National Socioeconomic Characterization Survey [19]. These regions are also characterized by a high proportion of rural population and reduced job opportunities, which limit access to adequate and sufficient food [20,21]. In addition, the significant proportion of the indigenous population in these areas adds a layer of overall vulnerability [22,23].

Unemployment was another factor found to be strongly associated with food insecurity in our research. Women who did not have a job were much more likely to be food insecure (OR = 1.27; 95% CI: 1.02–1.58). This finding is consistent with the international literature. For instance, in the case of Venezuelan migrants in Peru, Al-kassab-Córdova et al. (2023) found a direct association between unemployment and moderate and severe food insecurity, with particularly high prevalences in those who also lacked health insurance [24]. In Trinidad and Tobago, Saint Ville et al. (2022) noted that unemployed Venezuelan migrants were more than twice as likely to be in a status of generalized food insecurity than their fellow countrymen who were employed (67.6% vs. 56.7%), with a significant reduction in employment-related risk (adjusted OR = 0.112; 95% CI: 0.016–0.763) [25]. In addition, a longitudinal study conducted in the United States by Raifman et al. (2021) during the pandemic reported that the receipt of unemployment insurance was associated with a 35% decline in food insecurity among people who had lost their jobs, especially when they were receiving a federal supplement of $600 a week [26]. Similarly, in Ethiopia, Etana and Tolossa (2017) found that households headed by unemployed people had significantly higher levels of food insecurity, demonstrating the close relationship between access to work and food purchasing power [27]. The USDA report also confirmed that domestic unemployment is one of the most consistent predictors of increased food insecurity in the United States, along with inflation and the relative price of food [28].

This pattern aligns with what has been described in the literature as the feminization of food insecurity, in which gendered inequalities, caregiving roles, and lower accumulation of economic resources heighten women’s vulnerability [29]. Among immigrant women, this situation is further intensified by forms of structural exclusion linked to limited access to services, social protection, and stable employment opportunities [2], reinforcing the intersectional nature of the mechanisms observed.

This pattern underscores the structural dimension of food security as being closely associated with exclusion from the labor market, a situation associated with reduced opportunities to access formal and steady occupations with social protection. Therefore, gender-specific employment policies, as well as grants directed at unemployed women, should be a component in the design of integrated interventions, recognizing that employment could be a determinant of food security and family health.

Major discrepancies are evident when comparing the prevalence of food insecurity in immigrant women with national and international statistics. On the one hand, the prevalence reported in this study exceeded that reported by Greenwald and Zajfen (2015) in immigrants in the United States (20.1%) and by Kamelkova et al. (2023) in Syrian refugees resettled in Norway (22%) [30,31]. On the other hand, it was lower than that found by Vahabi et al. (2013) in Latin American immigrants in Canada (56%), as well as than that reported by Deschak et al. (2022) in immigrants in Mexico (75%) and, locally, than the findings by Maury-Sintjago et al. (2019), who reported a prevalence of 78% in the Haitian population residing in southern Chile [32,33,34]. This variability suggests that food insecurity in migrant populations depends on multiple contextual factors such as social inclusion processes and the functioning of institutional support networks, among others.

Furthermore, the comparison between the two groups reveals that immigrant women have a significantly higher prevalence of food insecurity than Chilean women. The difference increases when disaggregating data according to food insecurity levels, with a particularly marked difference being noted in the severe level (Chileans: 9.2% vs. immigrants: 18.1%). Our findings confirm the close relationship between migration and food insecurity, showing that immigrant status was associated with nearly threefold higher odds of experiencing this problem when compared to local citizens. This association highlights immigrant status as a structural determinant that limits access to a healthy diet, primarily in contexts of social and economic vulnerability [35,36].

Among Chilean women, the protective effect of age may reflect advantages accumulated across the course of life, particularly the strengthening of social ties and a greater psychological capacity to cope with stressful situations, both of which may help mitigate food insecurity [37,38]. In contrast, immigrant women—many of whom have shorter residence duration and more precarious labor conditions—may lack such protective resources, which attenuates the potential mitigating effect of age on food insecurity risk. Similarly, the stronger effect of unemployment observed among immigrant women may be related to their higher concentration in informal, unstable, and low-protection jobs, where job loss may have more severe consequences in contexts where unemployment insurance is absent [34,39].

Furthermore, the predictive model for all the participating women revealed that the risk of experiencing food insecurity is twice as high among women who belonged to lower socioeconomic statuses, were heads of household, had children, were unemployed, and had depression. In terms of socioeconomic status, this is a significant factor in global food insecurity, being much more prevalent among the poor. Indeed, a systematic review demonstrates that food insecurity is a hallmark of extreme poverty contexts, with social and economic deprivation deeply intertwined with risks such as gender-based violence [40]. During 2021, this scenario affected 35.8% of households below the poverty line in the United States, compared to 5.1% of households with incomes above the poverty line [41]. In Mexico, 48.6% of the households were in the low quintile, and in Canada, the odds of reporting food insecurity were 4.5 times higher among people living below the poverty line [42,43]. In Latin America, 28.2% of the population faces food insecurity, which is concentrated in the most vulnerable groups [4]. In Chile, national statistics have shown that 40.1% of the poorest households experience food insecurity, compared to only 8.1% of the richest households [18].

This disparity suggests that widespread poverty could be a structural factor that determines the lack of access to sufficient quality food. Education also emerges as a key determinant, as higher educational attainment has been associated with better preparedness to cope with adverse conditions [44]. However, in the south-central Chilean context, its protective effect may be limited by structural labor market inequalities that disproportionately affect immigrant women.

In addition, our results show that having children and being a head of a household are factors consistently associated with an increased risk of food insecurity (FI), both in the total sample and in the subgroups by nationality. In the overall multivariate model, the presence of children and being the head of the household increased the odds of FI by 82% and 53%, respectively. Among immigrant women, having children doubled the risk of FI, and being the head of the household increased it by 39%. In Chilean women, associations were even more pronounced in both variables. These findings are in line with the international literature. The risk of experiencing FI increases more than threefold among those who have children (OR = 3.6; 95% CI: 1.6–7.4), according to a study conducted in southern Chile among the Haitian community [33]. Additionally, in a study on Libyan migrants residing in Australia, household size (as an indirect proxy for motherhood) increased the risk of FI by 21% for each additional member (OR = 1.21; 95% CI: 1.01–1.47) [45]. Furthermore, Jung et al. (2016) conducted a meta-analysis of 42 studies, including more than 230,000 people, and found that female-headed households were 75% more likely to be food insecure (OR = 1.75; 95% CI: 1.55–1.98) [46].

Our results support a strong association between food insecurity and depression symptoms, demonstrating that women living with food insecurity were 2.26 times more likely to show depressive symptoms compared to those living in food-secure households (OR = 2.26; 95% CI: 1.68–3.04). This is consistent with several studies conducted worldwide. For example, Reeder et al. (2022), in their study, including data from the NHANES 2005–2016, found that adults with very low food security had 3.5 times more odds of suffering from depression (OR = 3.50; 95% CI: 2.98–4.12) compared to fully food-secure adults [47]. Similarly, a meta-analysis by Arenas et al. (2019) showed a strong association between FI and depression (OR = 2.74; 95% CI: 2.52–2.97), being one of the most frequent effects observed with respect to mental health conditions in adults [48]. Although other analyses had shown more moderate associations, the evidence remains consistent: a meta-analysis by Pourmotabbed et al. (2020), which included 372,143 participants from ten countries, estimated an overall OR of 1.40 (95% CI: 1.30–1.58), with a higher risk in older adults [49]. It is also important to highlight that the relationship between food insecurity and depression is bidirectional, and food insecurity has been consistently associated with psychological distress, while depressive symptoms may undermine functional capacity and economic stability, thereby reinforcing a self-perpetuating cycle of vulnerability [48,49].

This relationship has also been documented in populations of greater psychosocial vulnerability. In a study including refugees and immigrants in South Africa, Maharaj et al. (2017) found that not having sufficient food was associated with a more than fourfold increase in the risk of depression (OR = 4.51; 95% CI: 2.01–10.09), while eating less due to a lack of resources doubled that risk (OR = 2.88; 95% CI: 1.54–5.39) [50]. Similarly, Weigel et al. (2007) found that food-insecure households of migrant farmworkers on the US–Mexico border reported higher rates of depressive symptoms, along with cultural manifestations of distress such as “nervios” or persistent sadness [51]. These results concur with those by Maynard et al. (2018), who observed a dose-dependent relationship between FI severity and symptoms of depression in women from high-income countries, particularly among mothers, migrants, and women who had been exposed to violence or structural poverty [52].

Evidence also shows that these effects are aggravated when other disadvantages accumulate. For example, a study of pregnant adolescents in Ghana reported that living in large family-sized households was associated with an increased likelihood of developing depression (AOR = 2.78; 95% CI: 3.98–10.19); moreover, the interaction between severe food insecurity and large family size significantly increased depressive symptoms [53]. Although this study focused on the adolescent population, its findings illustrate how the accumulation of family responsibilities in food-insecure contexts can profoundly affect women’s mental health. In the same vein, studies conducted in Toronto with women who use food banks showed how motherhood and household headship translate into extreme strategies such as skipping meals, sending children to eat at other people’s homes, or pawning assets in order to purchase food [7].

From a structural approach, these associations express what Ivers and Cullen (2011) have called the “feminization of food insecurity,” in which women, especially single mothers and migrants, absorb the emotional, economic, and physical cost of sustaining the household’s diet [54]. As documented in Canada and Pakistan, being the head of a household and having children operates as a “triple burden”, restricting access to resources, paid employment, support networks, and safe food [55,56]. This scenario also reflects the global paradox, in which the widespread availability of ultra-processed and low-nutrient foods coexists with nutritional deficiencies and heightened food insecurity, particularly among socioeconomically vulnerable groups [57].

Although there are discrepancies around the variables that have an effect on FI between Chilean women and immigrants, such as age and employment, there are other variables that have been identified as drivers in both population groups. These are low socioeconomic status, having children, and being the head of a household. In addition, an association between FI and depression has been observed in both population groups. This indicates consistency across populations, highlighting the importance of these factors in women of both groups.

Therefore, our findings must be understood from an intersectional perspective, where gender, motherhood, household structure, and migration are cumulative dimensions of vulnerability. The status of being the head of the household not only embodies an administrative position of the family unit, but also represents a structural overload in terms of economy and care, which often remains invisible in public policies. In this sense, and as Tarasuk (2001) has argued, food insecurity cannot be addressed solely in terms of the income dimension but also should be examined as part of the material, symbolic, and social conditions that shape women’s lives as household managers [7].

This study presents limitations that should be considered when interpreting the findings. First, the cross-sectional design, inherent to prevalence studies, restricts the ability to examine causal relationships. Second, biosociodemographic characteristics and health conditions, such as depressive symptoms, were obtained through self-reported measures, which may introduce recall or social desirability biases. Third, although immigrant women were analyzed as a single group to ensure statistical power and comparability, this population is internally diverse; therefore, future research should incorporate within-group assessments to capture relevant heterogeneity. Finally, the geographic focus on the Ñuble, Biobío, and Araucanía regions reflects an intentional sampling strategy aligned with the study’s objectives; accordingly, the results should be interpreted within this territorial context, characterized by marked socioeconomic vulnerability, rather than extrapolated to the national level.

In the Chilean context, the 2021 Migration and Foreigners Law (Law No. 21.325) established access to essential social services for migrants who meet residency requirements [58]. However, eligibility for emergency food assistance and municipal social protection programs often depends on formal registration processes that may be harder to complete for recently arrived or irregular migrants. These administrative barriers may reinforce the vulnerabilities identified in this study.

In this regard, and based on the risk factors identified in this study, strategies are recommended to help mitigate insecurity, such as subsidies to purchase basic food products and access childcare services; employment and training policies to facilitate incorporation into the labor market; psychological counseling centers; and nutrition education programs to promote healthy diets, cooking skills, and food supply.

## 5. Conclusions

There is a high prevalence of food insecurity among all the women studied, particularly among immigrants. In both groups of women, risk factors for food insecurity are related to structural determinants such as socioeconomic status, having children, and being the head of a household, in addition to suffering from depression.

## Figures and Tables

**Table 1:** Biosociodemographic characteristics of the participants by nationality.

Biosociodemographic Characteristics	Total (n = 2124)	Chilean (n = 1062)	Immigrant (n = 1062)	*p*
Age				
18–29	848 (39.9)	437 (41.2)	411 (38.7)	<0.001
30–39	754 (35.5)	290 (27.3)	464 (43.7)	
40–49	522 (24.6)	335 (31.5)	187 (17.6)	
Socioeconomic status				
High/Medium-high	580 (27.3)	322 (30.3)	258 (24.3)	0.002
Medium-low/Low	1544 (72.7)	740 (69.7)	804 (75.7)	
Partner				
Yes	1380 (65.0)	776 (73.1)	604 (56.9)	<0.001
No	744 (35.0)	286 (26.9)	458 (43.1)	
Children				
Yes	1139 (56.6)	559 (52.6)	580 (54.6)	0.361
No	985 (46.4)	503 (47.4)	482 (45.4)	
Head of Household				
Yes	1081 (50.9)	433 (40.8)	648 (61.0)	<0.001
No	1043 (49.1)	629 (59.2)	414 (39.0)	
Educational attainment				
≥12 years	1804 (84.9)	922 (86.8)	882 (83.1)	0.015
<12 years	320 (15.1)	140 (13.2)	180 (16.9)	
Paid job				
Yes	1328 (62.5)	596 (56.1)	732 (68.9)	<0.001
No	796 (37.5)	466 (43.9)	330 (31.1)	
HBP				
Yes	476 (22.4)	214 (20.1)	262 (24.7)	0.013
No	1648 (77.6)	848 (79.9)	800 (75.3)	
T2DM				
Yes	404 (19.0)	207 (19.5)	197 (18.6)	0.580
No	1720 (81.0)	855 (80.5)	865 (81.4)	
High cholesterol				
Yes	385 (18.1)	189 (17.8)	196 (18.5)	0.693
No	1739 (81.9)	873 (82.2)	866 (81.5)	
Depression				
Yes	435 (20.5)	274 (25.8)	161 (15.2)	<0.001
No	1689 (79.5)	788 (74.2)	901 (84.8)	
Tobacco use				
Yes	487 (22.9)	297 (28.0)	190 (17.9)	<0.001
No	1637 (77.1)	765 (72.0)	872 (82.1)	

Data expressed as frequencies and percentages; chi-square test; T2DM (type 2 diabetes mellitus); HBP (high blood pressure).

**Table 2:** Level of food security of the participants.

Food Insecurity	Total	Chilean (n = 1062)	Immigrant (n = 1062)	*p*
Security	1292 (60.8)	751 (70.7)	541 (50.9)	<0.001
Mild	313 (14.7)	119 (11.2)	194 (18.3)	
Moderate	229 (10.8)	94 (8.9)	135 (12.7)	
Severe	290 (13.7)	98 (9.2)	192 (18.1)	

Data expressed as frequencies and percentages; chi-square test.

**Table 3:** Risk factors for food insecurity among the participants.

Variables	Crude OR	Adjusted OR
Immigrant	2.32 (1.94–2.78)	2.61 (2.15–3.17)
Low socioeconomic status	2.38 (1.92–2.95)	2.25 (1.77–2.87)
Having children	1.98 (1.66–2.37)	1.82 (1.49–2.23)
Head of household	1.94 (1.63–2.32)	1.53 (1.25–1.87)
Not having a job	1.54 (1.28–1.83)	1.27 (1.02–1.58)
Suffering from depression	2.35 (1.77–2.96)	2.11 (1.66–2.67)

Multivariate binary logistic regression.

**Table 4:** Risk factors for food insecurity in the participating women according to the study group.

	Chilean		Immigrant	
Variables	Crude OR	Adjusted OR	Crude OR	Adjusted OR
Age	1.01 (0.98–1.02)	0.97 (0.95–0.99)	NI	NI
Low SES	3.08 (2.11–4.50)	2.94 (2.01–4.31)	2.45 (1.87–3.22)	1.95 (1.43–2.65)
Having children	1.76 (1.19–2.61)	1.73 (1.18–2.54)	2.39 (1.87–30.7)	2.11 (1.62–2.75)
Head of household	1.99 (1.52–2.61)	1.93 (1.42–2.79)	1.49 (1.16–1.91)	1.39 (1.07–1.82)
Not having a job	NI	NI	2.15 (1.65–2.82)	1.33 (1.01–1.83)
Depression	2.31 (1.64–3.81)	1.83 (1.34–2.51)	2.19 (1.54–3.10)	2.09 (1.44–3.05)

Multivariate binary logistic regression. NI: Not included (*p* > 0.05).

## Data Availability

The original contributions presented in this study are included in the article. Further inquiries can be directed to the corresponding authors.
